# Cognitive Mechanisms Underlying Directional and Non-directional Spatial-Numerical Associations across the Lifespan

**DOI:** 10.3389/fpsyg.2017.01421

**Published:** 2017-08-23

**Authors:** Manuel Ninaus, Korbinian Moeller, Liane Kaufmann, Martin H. Fischer, Hans-Christoph Nuerk, Guilherme Wood

**Affiliations:** ^1^Leibniz-Institut für Wissensmedien Tübingen, Germany; ^2^Department of Psychology, University of Graz Graz, Austria; ^3^LEAD Graduate School and Research Network, Eberhard Karls University of Tübingen Tübingen, Germany; ^4^Department of Psychiatry and Psychotherapy A, General Hospital Hall, Austria; ^5^Division of Cognitive Sciences, Department of Psychology, University of Potsdam Potsdam, Germany; ^6^Department of Psychology, Eberhard Karls University Tübingen Tübingen, Germany

**Keywords:** SNARC effect, spatial-numerical bias, line bisection task, cognitive development, aging

## Abstract

There is accumulating evidence suggesting an association of numbers with physical space. However, the origin of such spatial-numerical associations (SNAs) is still debated. In the present study we investigated the development of two SNAs in a cross-sectional study involving children, young and middle-aged adults as well as the elderly: (1) the SNARC (spatial-numerical association of response codes) effect, reflecting a directional SNA; and (2) the numerical bisection bias in a line bisection task with numerical flankers. Results revealed a consistent SNARC effect in all age groups that continuously increased with age. In contrast, a numerical bisection bias was only observed for children and elderly participants, implying an U-shaped distribution of this bias across age groups. Additionally, individual SNARC effects and numerical bisection biases did not correlate significantly. We argue that the SNARC effect seems to be influenced by longer-lasting experiences of cultural constraints such as reading and writing direction and may thus reflect embodied representations. Contrarily, the numerical bisection bias may originate from insufficient inhibition of the semantic influence of irrelevant numerical flankers, which should be more pronounced in children and elderly people due to development and decline of cognitive control, respectively. As there is an ongoing debate on the origins of SNAs in general and the SNARC effect in particular, the present results are discussed in light of these differing accounts in an integrative approach. However, taken together, the present pattern of results suggests that different cognitive mechanisms underlie the SNARC effect and the numerical bisection bias.

## Introduction

Over the last decades, different effects ascribable to spatial-numerical associations (SNAs) have been described (for reviews see Fischer and Shaki, 2014; Winter et al., 2015). These include, amongst others, spatial biases observed in number magnitude comparison or parity judgment tasks (e.g., Dehaene et al., 1993), line and string bisection tasks with numerical displays(Fischer, 2001b; de Hevia et al., 2006), a bias in numerical interval bisection tasks (Priftis et al., 2006; Zorzi et al., 2002) and biased mental arithmetic (e.g., McCrink et al., 2007; Knops et al., 2014; Shaki et al., 2017). They also include number-related effects on pointing and grasping performance (e.g., Fischer, 2003; Andres et al., 2004), on visual detection (e.g., Fischer et al., 2003; Galfano et al., 2006; Ristic et al., 2006; Salillas et al., 2008; Stoianov et al., 2008) and on digit writing (Perrone et al., 2010). Although a lot of research has been devoted to SNAs, no consensus has been reached regarding their origin. In this context, it is of particular interest to establish whether different SNAs draw on the same cognitive underpinning.

In an attempt to investigate this question Cipora et al. (2015) suggested a taxonomy to classify SNAs based on their spatial attributes (extension vs. directionality) and their numerical attributes (cardinality, interval, ordinality, operations). One of the most basic distinctions made by Cipora et al. (2015) is between directional SNAs and non- directional SNAs. An example of directional SNAs are faster responses to small numbers with left-side responses and to larger numbers with right-side responses, known as the “SNARC-effect” (e.g., Dehaene et al., 1993). An example of non-directional SNAs are general biases toward the position of the larger number – left or right – as was observed in line bisection with task-irrelevant flanker numbers (e.g., Experiment 2 of Fischer, 2001b). This central theoretical distinction between non-directional vs. directional associations (see also Patro et al., 2014) may also imply distinct origins/ cognitive underpinnings of the respective SNAs. However, this proposal remains to be evaluated empirically.

Recently, Fischer and Brugger (2011, see also Fischer, 2012; Myachykov et al., 2014) argued that three different possible origins of SNAs may be differentiated. According to the authors the mental representation of numerical magnitude information is influenced by (i) general principles in the physical world, such as gravity; (ii) sensory and motor interactions we experience and perform; and (iii) current task constraints on information processing (Fischer and Brugger, 2011). These three hierarchically related levels of influence on numerical representations were referred to as “groundedness,” “embodiedness” and “situatedness” of cognition, respectively (Fischer, 2012) – with all three of these jointly determining the strength of SNAs (for a recent review see Winter et al., 2015).

**FIGURE 1:**
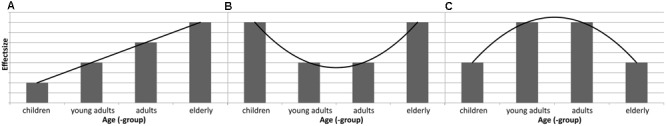
**(A)** Linear trend of increasing SNAs (*y*-axis) over the lifespan (*x*-axis), i.e., children, young adults, middle-aged adults, and elderly; **(B)** Positive quadratic trend of SNAs over the lifespan; **(C)** Negative quadratic trend of effect sizes of SNAs over the lifespan.

In our view, this hierarchical account of the representation of number magnitude and its implications for SNAs provide a testing bed to investigate the origin of different SNAs. In particular, it allows for rather specific predictions on how certain SNAs may manifest over the lifespan (see **Figure 1A**; e.g., the increasing influence of embodied number representations via cultural variables over the lifespan). Accordingly, the current study set out to evaluate whether the central theoretical distinction (non-directional vs. directional) of Cipora et al.’s (2015) taxonomy of SNAs may reflect distinct origins of the respective SNAs. Therefore, we selected two SNAs that differ with regard to directional vs. non-directional extension: According to Cipora et al. (2015) the SNARC (spatial-numerical association of response codes) effect is a directional SNA, reflecting that number magnitude is represented on a left-to-right oriented mental number line (MNL) with small numbers on the left and larger numbers represented toward their right side. Accordingly, faster responses are observed for congruent associations (i.e., left-small/right-large) than for incongruent ones (i.e., right-small/left-large; for reviews see Fias and Fischer, 2005; Gevers and Lammertyn, 2005; Hubbard et al., 2005; Wood et al., 2008; Fischer and Shaki, 2014).

Other studies found evidence for non-directional SNAs. For example, Fischer (2001b, Experiment 2) designed a version of the line bisection task in which two Arabic numbers presented near the left and right endpoints of horizontal lines served as flankers. When the larger flanker was on the left, a leftward bisection bias was observed, whereas a rightward bisection bias was found when the numerically larger flanker was on the right. Thus, midpoint estimation was distorted by task-irrelevant semantic activity induced by the number symbols (see also de Hevia et al., 2006; Bonato et al., 2008; de Hevia and Spelke, 2009; but see also Gebuis and Gevers, 2011).

Recent evidence also suggested that working memory may be a source of SNAs (Fias et al., 2011; van Dijck and Fias, 2011; Ginsburg et al., 2014). More specifically, van Dijck and Fias (2011) argued that the SNARC effect reflects an association between the ordinal position of an item in working memory and response side. In line with this argument, van Dijck et al. (2009) observed that the SNARC effect disappeared under working memory load. Thus, the SNARC effect may not necessarily reflect overlearned cultural and thus long-term associations between number magnitude and physical space but may, at least partly (cf. Huber et al., 2016), be constructed *ad hoc* during task execution as well, thus reflecting the situated origin of this SNA.

Examining variations in spatial-numerical performance over the lifespan may give us new insight into the origin(s) of both directional and non-directional SNAs as introduced above (for a similar approach, see Lambrechts et al., 2013). For instance, when the SNARC effect reflects an *ad hoc* association between the ordinal position of an item in working memory and the response side (van Dijck and Fias, 2011), the effect should be relatively smaller for children as well as the elderly when compared to young and middle-aged adults, due to working memory limitations (see **Figure 1C**). Specifically, a smaller working memory capacity, in terms of reduced memory spans and thus fewer numbers/ordinal positions maintained in working memory, might lead to smaller SNARC effects. This prediction is due to well-known age-related changes in working memory capacity over the life-span: generally, performance on working memory tasks is much better in adults as compared to children and the elderly (e.g., Hasher and Zacks, 1988; Siegel, 1994; Borella et al., 2008). However, if the SNARC effect instead reflects influences of embodied representations then one may expect a positive association of the SNARC effect with age (as observed in the meta-analyses by Wood et al., 2008) – reflecting an age-related strengthening of SNAs through longer experiences of culturally mediated sensory-motor constraints (for reviews see Fischer and Brugger, 2011; Fischer, 2012; see **Figure 1A**). In particular, when cognitive capacities become limited in the elderly, embodied cognition effects driven by the reactivation of previously built associations seem to get more pronounced (e.g., Engelen et al., 2011; Dekker et al., 2014; Loeffler et al., 2016 for an overview). In line with this argument a recent study (Hoffmann et al., 2014) indicated that the SNARC effect was more pronounced in elderly as compared to young adults.

For the case of the non-directional SNA measured with the line bisection task similar competing predictions can be derived: On the one hand, if the bias observed in line bisection is due to embodied mechanisms such as sensory-motor associations of larger magnitudes (e.g., Perrone et al., 2010) one would expect an age-related strengthening; this means a positive association between bisection bias and age (see **Figure 1A**). On the other hand, working memory might be an important predictor of estimation biases as well. More specifically, inhibitory control, which is seen as a specific part of working memory (for a review see Diamond, 2013) might influence participants’ bisection biases as it helps to ignore the task-irrelevant numerical flankers. Hence, weaker inhibitory capacity, as observed in children and elderly participants (e.g., Hasher and Zacks, 1988; Christ et al., 2001), should increase semantically driven bisection biases (see **Figure 1B**). Interestingly, Hoffmann et al. (2014) also attributed the stronger SNARC effect they observed in their elderly as compared to middle-aged adult participants to reduced inhibitory control over task-irrelevant associations (in this case of numbers and space) in older age (e.g., Hasher and Zacks, 1988). As such, the latter prediction of more pronounced SNAs in children and elderly may also apply to the SNARC effect.

Interestingly, the literature suggests differing developmental trajectories of the SNARC effect and the numerical bisection bias. Consider first the SNARC effect. Depending on the task the SNARC effect can be reliably observed in 7-year-old children (van Galen and Reitsma, 2008, but see Berch et al., 1999, for a SNARC effect from the age of 9 years on only). This suggests that the SNARC effect indeed increases with age. However, one might also speculate that the SNARC effect cannot be measured reliably in children younger than 7 years of age. Nonetheless, Hoffmann et al. (2013) already observed a SNARC effect in a magnitude classification as well as a color judgment task in kindergarten children. Moreover, a parity SNARC effect was already reported for Chinese preschoolers (Yang et al., 2014). Patro and Haman (2012) even observed a SNARC-like effect in 3- to 4-year-olds in a numerosity comparison task. Finally, Bulf and colleagues noticed directional left-to-right mappings in 8-month-old infants (see McCrink and Opfer, 2014 for a review on the development of SNAs; see Newcombe et al., 2015, for a review on the intertwined development of spatial and numerical competences). Apart from that, and consistent with an age-related increase of the SNARC effect, Wood et al. (2008) found an age-related increase in the SNARC effect size in a meta-analysis. Finally, the SNARC effect is preserved in neurological patients with visuospatial hemineglect even when spatial processing and other SNAs are distorted (Priftis et al., 2006), implying a considerable strength of the effect in this biologically oldest population. In summary, the SNARC effect reflects a robust SNA based on directional spatial representation, which can be found early in life, increases its effect size with age and seems to be preserved in the presence of visuospatial impairments (see **Figure 1A**).

Consider now the developmental trajectory of bisection biases. On simple line bisection, children between 4 and 12 years showed a clear shift from an initial rightward to a later leftward bias when instructed to bisect lines printed on a sheet of paper (see also Dellatolas et al., 1996). A leftward bisection bias was already found at the age of 7 that decreased slightly up to the age of 12 (Van Vugt et al., 2000). In young adults a systematic leftward bisection bias has been observed (Orr and Nicholls, 2005) whereas a stronger rightward bias was found in elderly participants compared to middle-aged and young adults (Fujii et al., 1995; see also Jewell and McCourt, 2000 for a meta-analysis). So far, there is only one study which compared children and adults regarding a numerical version of the line bisection task (de Hevia and Spelke, 2009). Importantly, de Hevia and Spelke (2009) investigated the numerical bisection bias in young adults as well as 5- and 7-year-old children. While adults presented with a robust bias toward the larger number in both symbolic (i.e., Arabic numbers) and non-symbolic (i.e., dots) flanker conditions, 5- and 7-year-old children only showed this bias for non-symbolic flankers. In sum, these results suggest a visuospatial as well as a (non-symbolic) magnitude bias in children and that these biases increase with age. However, effects of more advanced age on this spatial non-directional representation remain to be studied.

Taken together, the question of whether directional and non-directional SNAs (as for instance the SNARC effect and numerical bisection biases) have a common origin or not remains unanswered so far. On the one side, one may argue that a small set of cognitive processes should account for SNAs observed in different tasks. When several SNAs can be attributed to a common set of cognitive mechanisms (e.g., influences of inhibitory control, see above), one would expect that spatial-numerical biases obtained in different tasks should show a non-zero correlation. Moreover, the developmental trajectories of different SNAs should be relatively similar in this case. On the other side, different types of SNAs, e.g., those bound to spatial directions, such as the SNARC effect, or those bound to spatial extensions, such as the numerical bisection bias (see Cipora et al., 2015), may originate from different cognitive processes (e.g., Fischer and Brugger, 2011; Myachykov et al., 2014). In particular, (i) when the SNARC effect is linked to the strength of sensory and motor experiences and thus embodied representations of numbers one would expect a linear relation between the size of the SNARC effect and age (**Figure 1A**), given that embodied sensory-motor associations underlying the effect would get stronger with age. Moreover, in case the numerical bisection bias originates from the same process, a similar relation between the size of the bias and age should be found (**Figure 1A**). (ii) However, when the SNARC effect is linked to working memory capacity, one should observe a smaller size of the SNARC effect for both children and elderly than for young adults, because of well-known age-related changes in working memory capacity over the life-span (**Figure 1C**). In contrast, age related change of working memory capacity should not affect the size of the numerical bisection bias. (iii) Last, in case the SNARC is linked to the strength of inhibitory control abilities, one should observe a larger SNARC effect for both children and elderly adults than for young adults (**Figure 1B**). Similarly, when the numerical bisection bias originates from the same process, one might expect a larger bisection bias for both children and elderly as compared to adults (**Figure 1B**). However, as argued above, we do not hypothesize SNARC and bisection bias to originate from the same underlying processes. Instead, the SNARC effect may be driven by sensory and motor experiences whereas numerical line bisection biases may mainly originate from situated aspects of our cognition (e.g., *ad hoc* effects of inhibitory control). In this case, no significant correlations between these two SNAs should be found and the developmental trajectories of SNAs should be different across the lifespan. In the present study, we tested these predictions on the existence of shared cognitive mechanisms underlying distinct SNAs in a cross-sectional study involving participants between 9 and 86 years of age. In particular, we investigated the developmental trajectories of the SNARC effect and numerical bisection biases as well as the correlations between these SNAs.

## Materials and Methods

### Participants

Four groups of participants were included in the present study with an overall N of 100: 24 children, 25 young adults, 27 middle-aged adults and 24 elderly adults. All participants had normal or corrected to normal vision. Participation in the study was voluntary. This study was carried out in accordance with the recommendations of the institutional guidelines of the University of Salzburg and of the Declaration of Helsinki. Informed written consent was obtained from all participants or, in case of children, from their parents or caregivers prior to the study. The protocol was approved by the local ethics committee.

#### Children

Twenty-four right-handed third-grade children participated in the study (mean age = 9y1m, *SD* = 0y3m, range 8y6m to 9y8m). Handedness of all participants was assessed with the Edinburgh Handedness Inventory (Oldfield, 1971). Please note that we chose that particular age of our children sample because reliable SNARC effects were previously observed for children of this age or even younger (e.g., Berch et al., 1999, for 9-year-olds; van Galen and Reitsma, 2008, for 7-year-olds; Patro and Haman, 2012, for 4-year-old children using non-symbolic number tasks; Shaki et al., 2012; McCrink et al., 2014, SNARC like effects in counting direction of 3–4 year olds; Bulf et al., 2016, in 8–9-month-old infants).

#### Young Adults

Twenty-five young adults, all except one being right-handed^1^, were examined (mean age = 21y7m; *SD* = 1y10m; range 18y2m to 26y5m). All participants completed at least 12 years of general schooling or a specific technical training.

#### Middle-Aged Adults

Twenty-five right-handed and two ambidextrous middle-aged adults were examined (mean age = 46y0m; SD = 4y1m; range 36y4m to 52y0m).

#### Elderly

Twenty-four community-dwelling elderlies, all except one being right-handed, were tested (mean age = 67y0m; SD = 6y3m; range 60y1m to 86y5m). According to self-report, all elderly participants were free from major neurological and psychiatric diseases.

### Stimuli, Design and Procedure

#### Parity Judgment Task

Arabic digits 1, 2, 8, and 9 were presented on a CRT monitor in white on a black background. Participants decided whether Arabic digits were odd or even by pressing a right or left response key (i.e., right and left Ctrl keys of a standard keyboard). Arabic digits were shown in Arial font size 50 for a maximum of 2000 ms and covered a visual angle of 2.5° vertically and 2° horizontally from a viewing distance of 50 cm. A fixation cross “+” in the middle of the computer screen was presented in the inter-stimulus interval for 1000 ms on average (range: 400 – 1600 ms). Response keys (12 by 12 mm) were positioned in front of the participant and were 16 cm apart. Reaction time (RT) was recorded for a maximum of 2000 ms after stimulus presentation.

The assignment of response keys to even or odd numbers was counterbalanced across participants: After half of the experiment, the parity-to-response-key assignment was reversed (from even-right/odd-left to even-left/odd-right or vice versa). Each stimulus was repeated 40 times per parity-to-response-key assignment, resulting in a total of 320 experimental trials. Additionally, ten practice trials, which were not considered for analyses, preceded the experimental trials in each parity-to-response-key assignment. In total the experiment took participants approximately 20 min to complete.

The high number of repetitions of individual stimuli was chosen to ensure sufficient reliability (following the recommendation of Cipora and Nuerk, 2013). To balance the occurrence of individual stimuli, these were presented in 4 blocks of 40 trials for each half. Within each block each stimuli occurred 10 times. Accordingly, split-half reliability was computed by correlating z-transformed SNARC slopes from odd and even blocks across participants for each group separately. Spearman-brown corrected split-half reliability coefficients were: *r*_children_ = 0.76, *r*_young adults_ = 0.93, *r*_middle-aged adults_ = 0.95, *r*_elderly_ = 0.96.

#### Line Bisection Task

A numerical and a non-numerical version of this task were employed. In the numerical version, participants were asked to precisely bisect 16 horizontal lines; eight lines were printed on each of two A4 sheets of paper and flanked by two Arabic digits. The sheets of paper were positioned in the mid-sagittal plane in front of participants in portrait orientation. Eight lines of 145 mm length were printed with different horizontal offsets from each other on each of the two pages and with a vertical separation of 28 mm between them. Numerical distance between flankers was manipulated: digit pairs 1/1, 2/2, 8/8 and 9/9 were presented in the *symmetrical flankers* condition to investigate a possible *magnitude-based numerical bias*, and digit pairs 1/2, 2/1, 8/9 and 9/8 were presented in the *asymmetrical flankers* condition to evaluate a possible *difference-based numerical bias*. Digits were placed close to the start and endpoint of the lines and were printed in boldface 18 point Monaco font.

In the non-numerical version, the only difference to the numerical version of the task was that the flankers were omitted from each line. Importantly, the spatial position of lines on the two sheets in the non-numerical version of the line bisection task was the same as in the numerical version. This allowed for a direct comparison of homolog trials in both numerical and non-numerical tasks. Order of presentation of the numerical and non-numerical task version was counterbalanced between participants. For reasons of consistency, we refer to the homologue trials in the non-numerical tasks (symmetrical and asymmetrical conditions but with no actual flankers) also as if they would exhibit *magnitude-based* and *difference-based spatial bias*, respectively, although these tasks were visually identical.

Reliability of the line bisection bias was analyzed separately for the numerical and non-numerical version of the task and for each age group. In particular, split-half reliability scores were computed by correlating performance in two halves of the task matched for the occurrence of *asymmetrical* and *symmetrical flankers* (i.e., items 2/2, 2/1, 9/9, 8/9 vs. 1/1, 1/2, 8/8, 9/8; corresponding items in the non-numerical version) across participants of the respective age group. Spearman-brown corrected split-half reliabilities were as follows for numerical, *r*_children_ = 0.69, *r*_young adults_ = 0.91, *r*_middle-aged adults_ = 0.82, elderly = 0.92, and non-numerical version of the task, *r*_children_ = 0.71, *r*_young adults_ = 0.94, *r*_middle-aged adults_ = 0.63, *r*_elderly_ = 0.73).

## Results

### Parity Judgment Task

RT data was trimmed prior to statistical analyses. Correct responses slower than 200 ms contained in the interval defined by ±3 standard deviations from the individual mean RT were kept while responses outside this interval were excluded from analyses. This procedure was repeated iteratively for each individual until no more responses were excluded (5% excluded on average). Because of considerable differences in average RT across groups, which may mask differences between groups, we z-standardized RT using individual means and standard deviations (see Faust et al., 1999 for the statistical rationale). Mean RT as well as the absolute and z-transformed SNARC slopes computed from RT per participant (Lorch and Myers, 1990) served as dependent variables (see **Table 1** for descriptive statistics). SNARC slopes describe the best-fitting linear regression of RT difference scores (RT right hand minus RT left hand) on digit magnitudes (see Fias et al., 1996 for a more detailed description of the estimation procedure). Hence, faster right-handed responses to larger numbers result in negative SNARC slopes. Errors were infrequent and will not be analyzed separately (4% excluded on average).

**Table 1:** Age, average reaction times as well as the standardized and non-standardized SNARC slopes in the parity decision task.

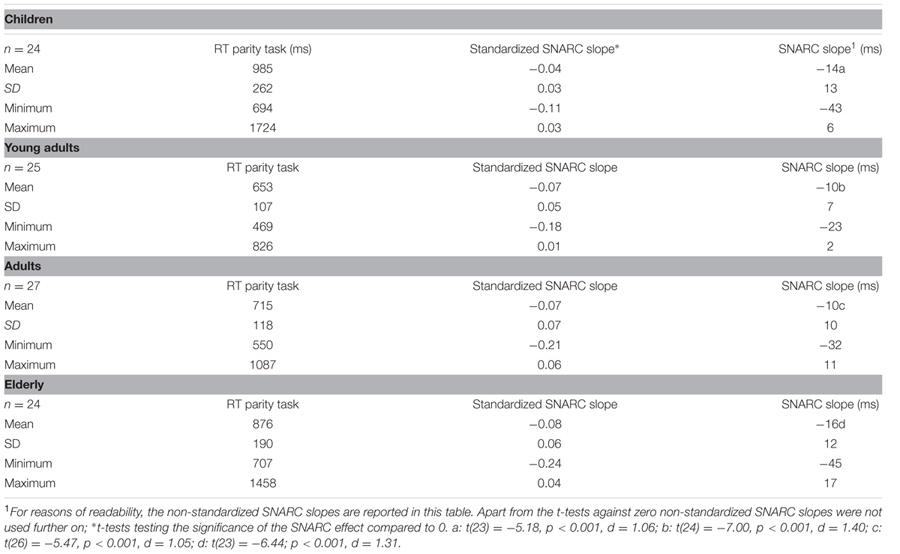

#### Mean RT

Mean RT differed significantly between groups [*F*(3, 96) = 17.77; *MSE* = 31.747; *p* < 0.05, η^2^ = 0.36]. Bonferroni-corrected pairwise-comparisons revealed comparable response latencies for children and elderly participants (*p* = 0.23) on the one side and for young adults and middle-aged adults (*p* > .99) on the other side (see also **Table 1**). Moreover, children (*d* = 1.67, *d* = 1.35) and elderly participants (*d* = 1.46, *d* = 1.03) were significantly slower than young adults and middle-aged adults (both comparisons *p* < .05; i.e., children = elderly participants > middle-aged adults = young adults).

#### SNARC Effects

Significant SNARC slopes were observed for each age group, with means of -14, -10, -10, and -16 ms/digit for children, young and middle-aged adults as well as elderly, respectively (all *p* < .05; see **Table 1**). Due to significant differences in overall RT raw SNARC slopes in ms/digit were not used further in our analyses but z-transformed slopes.

Significant z-transformed SNARC slopes were obtained for each age group [children: *t*(23) = -5.44, *p* < 0.001, *d* = 1.11; young adults: *t*(24) = -7.32, *p* < 0.001, *d* = 1.46; middle-aged adults: *t*(26) = -5.48, *p* < 0.001, *d* = 1.05; elderly: *t*(23) = -6.42; *p* < 0.001, *d* = 1.31; see **Table 1**). In order to examine differences between age groups, z-transformed SNARC slopes were submitted to a one-way analysis of variance (ANOVA). A main effect of participant group indicated that standardized SNARC slopes differed significantly between groups [*F*(3,96) = 3.15, *MSE* = 0.003, *p* < 0.05, η^2^= 0.09]. Bonferroni-corrected pairwise-comparisons revealed a significant difference between children and elderly participants (*p* < 0.05, *d* = 0.91). Other pair-wise comparisons did not reach significance (all *p* > 0.16, see also **Table 1**). In each age group, a large proportion of participants presented a negative SNARC slope [22/24 children (92%), 21/25 young adults (84%), 23/27 middle-aged adults (85%) and 22/24 elderly participants (92%)].

### Line Bisection Task

We measured the constant error to the middle of the lines in millimeters, so that negative values reflected a leftward bias and positive ones a rightward bias. The response of one elderly participant to the *symmetrical flanker* digit pair 8/8 was replaced by the elderly participants group mean since the response deviated more than three standard deviations from the group mean and probably reflected a momentary lapse of attention.

The biases observed for *symmetrical* and *asymmetrical flankers* were analyzed in the numerical version (*magnitude-based* and *difference-based numerical bias*) of the line bisection task as well as for their counterparts in the non-numerical version (*magnitude-based* and *difference-based spatial bias*). These bias scores were submitted to a 2 × 4 ANOVA with the factors flanker magnitude (small vs. large [symmetrical]; large magnitude left vs. large magnitude right [asymmetrical]) and group (children, young adults, middle-aged adults, elderly) in the numerical version of the task.

For the non-numerical version of the task two univariate ANOVAs were conducted with the factor group (children, young adults, middle-aged adults, and elderly), one for the homologue items of the asymmetrical flankers task (*difference-based spatial bias*; mean of non-numerical items presented at the same position as the items with large magnitude on the left/right) and one for the homologue items of the symmetrical flankers task (*magnitude-based spatial bias*; mean of non-numerical items for large/small numbers). Moreover, *t*-tests against zero were conducted to evaluate the statistical significance of spatial biases in non-numerical line bisection tasks in the different age groups.

#### Symmetrical Flankers

The *t*-tests revealed no significant *magnitude-based spatial bias* in the non-numerical bisection task (all *p* > 0.47).

In the ANOVA, no main or interaction effects did reach significance in the numerical version of the line bisection task.

Also, the univariate ANOVA for the non-numerical version of the line bisection task yielded no significant effect (*p* = 0.93).

#### Asymmetrical Flankers

Again, the *t*-tests revealed no significant *difference-based spatial bias* in the non-numerical bisection task (all *p* > 0.10).

Additionally, the univariate ANOVA for the non-numerical version of the line bisection task did not yield a significant effect (*p* = 0.62)^2^, thus ruling out contributions to the above effects from stimulus order or positioning.

**FIGURE 2:**
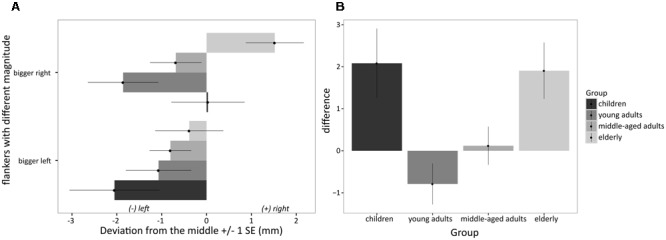
**(A)** *Difference-based numerical bias* observed in children, young adults, middle-aged adults and elderly participants for numerical flankers with differing magnitude illustrated for larger number on the left and larger number on the right separately (asymmetrical flankers); positive/negative numbers indicate a rightward/leftward bias, respectively; **(B)** *Difference-based numerical bias* observed in the different age groups for numerical flankers with different magnitude illustrated as general bias toward larger numbers independent of side; positive difference values thus indicate a bias toward larger numbers, i.e., the larger the difference the larger is the bias toward the larger number.

In the numerical version of the task, the ANOVA revealed a significant main effect of side of the larger number [*F*(1,96) = 7.20; *MSE* = 4.77; *p* < 0.05, $\eta_{p}^{2}$= 0.07; see also **Figure 2A**]: Mean estimations for larger numbers on the left (mean = -1.07; negative value indicates bias toward larger number) and larger numbers on the right (mean = -0.28; positive value indicates bias toward larger number) differed significantly with regard to the mean position of the estimates on the line. More specifically, mean estimations were located further to the left when the larger number was on the left as compared to the estimations when larger numbers were positioned on the right. Importantly, this main effect was qualified by group [*F*(3,96) = 5.05; *MSE* = 4.77; *p* < 0.05, $\eta_{p}^{2}$= 0.14]. No other main or interaction effect was significant. In a complementary analysis, planned comparisons were conducted on the difference between the bias obtained when the larger number was on the right vs. on the left. The difference larger number on the right minus larger number on the left was calculated for each person separately and submitted to an univariate ANOVA with the factor group. For this index, positive values indicate a bias toward the larger number (see **Figure 2B**). A significant effect group was observed [*F*(3,96) = 5.05; *MSE* = 9.54; *p* < 0.05, η^2^= 0.14]. Additionally, the univariate ANOVA revealed a significant positive quadratic trend of the data [*F*(3,96) = 14.04; *p* < 0.05, η^2^= 0.13], indicating a U-shaped relationship between age groups and *difference-based numerical bisection bias*. This trend reflects a higher bias toward larger numbers in children and elderly as compared to young adults and middle-aged adults (see **Figures 1B, 2B**). Bonferroni-corrected pairwise-comparisons indicated that a larger bisection bias was observed in children (*M* = 2.08 mm, *SD* = 4.05, *d* = 0.86) and elderly participants (*M* = 1.91 mm, *SD* = 3.30, *d* = 0.93) as compared to young adults (*M* = -0.79 mm, *SD* = 2.44; both *p* < 0.05). The bias observed in children, middle-aged adults (*M* = 0.12 mm, *SD* = 2.37), and elderly participants did not differ significantly (all *p* > 0.15), as was that observed for young and middle-aged adults (see **Figure 2B**).

### Age, SNARC and the Bisection Bias

**FIGURE 3:**
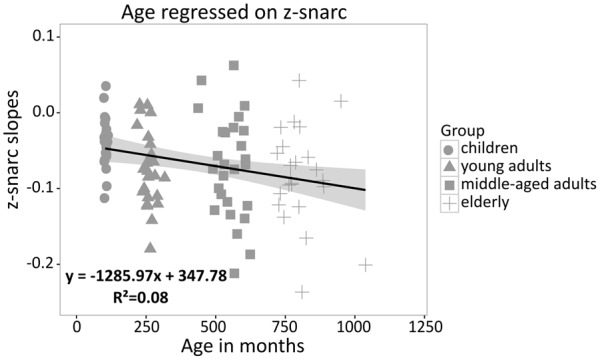
Individual z-standardized SNARC slopes regressed on age (children, young adults, middle-aged adults, elderly); the gray shade indicates a 95% confidence region for the regression fit.

In order to investigate whether a significant association between the SNARC effect and age was present, individual z-standardized SNARC slopes were regressed on age. The effect of age on the SNARC slope was small but, in line with our expectations and the meta-analytical results reported by Wood et al. (2008), the SNARC slope became significantly more negative with age [*R^2^* = 8%, β = -0.27, *t*(98) = -2.83, *p* = 0.006; see **Figure 3**].

In order to investigate the existence of a common mechanism underlying SNAs, correlations between age, the SNARC effect and the bisection bias were calculated. Results were very clear: Non-numerical bisection biases [*magnitude-based spatial bias* (i.e., mean of homologue conditions for small and large numbers) and *difference-based spatial bias* (i.e., mean of homologue conditions for larger number on the left and larger number on the right)] correlated moderately with each other but not with the SNARC effect (see **Table 2**). More importantly, numerical bisection biases [*magnitude-based numerical bias* (i.e., difference between larger vs. small numbers) and *difference-based numerical bias* (i.e., difference between larger number on the left vs. on the right)] did not correlate with the SNARC effect either. Finally, we assessed whether the relation between performance in the SNARC and bisection tasks was moderated by age. Thus, we examined the partial correlation between z-SNARC and bisection toward larger number, controlling for individuals age. However, the partial correlation was not significant (*r* = 0.08, *p* = 0.39) as well. This indicates that the correlation between directional (SNARC) and non-directional (bisection bias) SNAs may not be moderated by age in the present study. Thus, our results do not corroborate the notion of a common origin of these bisection biases and the SNARC effect.

**Table 2:** Correlations between age, SNARC slope and measures of *magnitude-based* and *difference-based bias* in the numerical and non-numerical versions of the line bisection task.

	Age	SNARC	Magnitude-based spatial bias	Magnitude-based numerical bias	Difference-based spatial bias	Difference-based numerical bias
age						
SNARC	–0.27^∗^					
Magnitude-based spatial bias	0.08	–0.05				
Magnitude-based numerical bias	–0.03	0.01	–0.16			
Difference-based spatial bias	0.08	–0.06	0.55^∗^	–0.02		
Difference-based numerical bias	0.03	0.07	–0.04	0.16	–0.07	

*^∗^*p* < 0.05.*

## Discussion

The aim of this study was to examine whether different SNAs share common or distinct cognitive origins. Based on Cipora et al.’s (2015) distinction between directional and non-directional SNAs, we investigated the SNARC effect in parity judgments (a directional SNA) and the spatial bias in bisecting lines flanked by digits (a non-directional SNA) over the lifespan. Evaluating the developmental trajectories of SNAs should provide meaningful information with respect to the question whether different SNAs share common cognitive underpinnings – as indicated by similar developmental patterns over the lifespan.

Results indicated a reliable SNARC effect in all age groups tested. Moreover, in line with the meta-analytical findings by Wood et al. (2008), we found the SNARC effect to increase with age across the lifespan (see also Hoffmann et al., 2014). As regards the bisection task, we observed a larger *difference-based numerical bias* in children and elderly as compared to young adults. However, different from the SNARC effect no linear effect of age on the bisection bias was observed, but a quadratic one. Finally, we did not observe a significant correlation between the SNARC effect and the line bisection bias. This observation, together with a specific effect of age on the SNARC effect, are in line with the view that different cognitive mechanisms seem to underlie the directional and non-directional SNAs, as reflected by the SNARC effect and the bisection bias, respectively. In the following, we will discuss these points in more detail.

### The SNARC Effect

A significant SNARC effect was found in all age groups, and its size increased as a function of age. Importantly, the latter finding cannot be accounted for by differences in processing speed because the increase in the size of the SNARC effect from childhood to old adulthood was found for z-transformed SNARC slopes, thus controlling for differences in absolute response latencies between groups (Faust et al., 1999). In general, these findings confirm the expected age-related increase in the SNARC effect (see **Figure 1A**) indicated by a meta-analysis (Wood et al., 2008). However, the association between the SNARC effect and age obtained in the current study was lower than the one previously reported in the meta-analysis. Most plausibly, this discrepancy may be due to differences in stimuli, procedures, and populations relative to the 17 studies considered by Wood et al. (2008). The same materials and procedure were, however, used in the present study to examine participants from different age groups; therefore, the observed results corroborate and strengthen the previous conclusion that the SNARC effect increases with age.

Theoretically speaking, the observed increase in the size of the SNARC slopes as a function of age might reflect increasingly automatic associations between number magnitude and physical space across the lifespan. Thereby, the present results are consistent with the role of embodied representations on a SNA bound to spatial directions such as the SNARC effect. In particular, the observed age-related increase of the SNARC effect may be driven by longer-lasting experiences of cultural influences such as reading and writing direction over the lifespan. As such, accumulating sensory-motor associations may let the SNARC effect grow stronger with age. Nevertheless, one has to acknowledge that the amount of such experiences may well differ across (adult) individuals, thus allowing for differently strong SNAs. However, generally speaking, in children, directional SNAs may not yet be as automatic as in older adult populations. Supporting this view, van Galen and Reitsma (2008) have shown that 7-year-old children only show a SNARC effect in tasks where number magnitude is task-relevant. Only when children were 9 years and older they also showed a reliable SNARC effect in tasks where number magnitude is task-irrelevant – suggesting an increasingly automatic association of number magnitude and physical space. However, Hoffmann et al. (2013) found a SNARC-like effect in a color judgment task in children as young as 5 and a half years. This might suggest that the absence of a parity SNARC effect in 7- as opposed to 9-year-old children in the study of van Galen and Reitsma (2008) was not due to a less developed automatic association of number magnitude and physical space at these earlier developmental stages (see also Bulf et al., 2016, who observed directional left-to-right mappings already in 8-month-old infants; and Patro and Haman, 2012, for a SNARC-like effect in 4-year-olds). Importantly, our results are not incompatible with these previous results *per se*, as we observed a significant SNARC effect in children as well. However, the main focus of our study was on relative differences in SNARC effects between age groups for which we found that SNARC effect sizes increased with age. Moreover, no SNARC effect was observed in illiterate adult populations (Zebian, 2005), emphasizing the role of the spatial experience of reading and writing direction in creating a SNA bound to spatial directions (see McCrink and Opfer, 2014 for a review).

Moreover, the developmental trajectory of the SNARC effect is also meaningful with regard to influences of working memory on the origin of this SNA. In this view, the SNARC effect would reflect an *ad hoc* association between the ordinal position of an item in working memory and the response side (van Dijck and Fias, 2011). Consequently, the SNARC effect should have been relatively lower for children and elderly persons as compared to young and middle-aged adults (**Figure 1C**), due to age-related changes in working memory (i.e., higher working memory capacity in adults as compared to children and the elderly; e.g., Hasher and Zacks, 1988; Siegel, 1994; Borella et al., 2008). This argument is supported by a recent study of randomization behavior across the life span which concluded that cognitive performance peaks at around 25 years of age (Gauvrit et al., 2017).

Alternatively, however, it might be possible that the tendency to code ordinal positions in working memory spatially and the tendency to use strategies involving working memory resources to solve the task increases with increasing age (**Figure 1A**). According to this view, the working memory account of the SNARC effect might be actually consistent with the finding that the SNARC effect increases with age (i.e., smallest effects for children, intermediate effects for young adults, and largest effects for elderly adults). However, the findings of van Dijck et al. (2009, diminished SNARC effect under working memory load) indicate that working memory resources (in terms of capacity) seem to be necessary to observe a SNARC effect. Accordingly, when working memory capacity declines in elderly, the SNARC effect should decrease rather than increase according to this view. Moreover, it seems rather implausible to assume reliance on increasingly resource-demanding strategies in persons showing an actual decline of cognitive capacities such as working memory due to aging. According to this, elderly people would rely increasingly on strategies requiring cognitive resources that decline, rather than culturally acquired strategies manifested in behavior. In line with this point, it was recently found that effects of embodied cognition driven by the reactivation of previously built associations seem to be stronger in the elderly (e.g., Engelen et al., 2011; Dekker et al., 2014; Loeffler et al., 2016 for an overview). Therefore, we suggest that the observed continuous increase of the SNARC effect over the lifespan is hard to reconcile with such working memory accounts on the SNARC effect. Instead, it corroborates the influence of embodied representations reflecting cultural experiences on long-term associations between number magnitude and physical space. Interestingly, though, the observed change of the size of the SNARC effect across the lifespan might also result from an increase of the tendency to code spatially the numbers from childhood to adulthood joint with a decrease of the ability to inhibit irrelevant information in older age. As such, this account would actually reflect an interplay between mechanisms discussed to influence SNAs on their own earlier. According to this view, the observed trend is consistent with different accounts of the origin of the SNARC effect (e.g. van Dijck et al., 2009, 2012; see also Cheung et al., 2015). These different accounts might even include the polarity correspondence hypothesis, according to which spatial biases result from a binary coding of number magnitudes as being large or small, followed by their association with space that was equally coded into right vs left, respectively (e.g., Proctor and Cho, 2006). Thus, the mastery of spatial language might underlie the observed age-related strengthening of the SNARC effect. In fact, it may by possible that different mechanisms account for developmental changes of, for instance, the SNARC effect.

### The Bisection Bias

In line with previous studies (Fischer, 2001b; Calabria and Rossetti, 2005; de Hevia et al., 2006) we observed no reliable spatial bias for the presence of *symmetric flankers*. In other words, no *magnitude-based numerical bias* was observed in the numerical version of the bisection task. Furthermore, no differences between age groups were observed for the *magnitude-based numerical bias* but rather strong within-group variability. This suggests that the *magnitude-based numerical bias* is less systematic than other forms of numerical bias and reinforces the view that processing number magnitude alone may not be sufficient for eliciting a numerical bias (Calabria and Rossetti, 2005; de Hevia et al., 2006) or biases for both sides were identical and canceled each other (Fischer, 2006). Moreover, the strong within-group variability observed in this study also reinforces the conclusion that the *magnitude-based spatial bias* may not be particularly robust. Finally, in line with previous studies, the present results suggest that the familiarity with numbers, which presumably increases with age as a result of lifelong handling of numbers (**Figure 1A**), is not sufficient for producing a stronger *magnitude-based numerical bias* in elderly participants (relative to children).

On the other hand, a robust *difference-based numerical bias* was found in the present study following the presentation of *asymmetrical flankers*. The significant main effect of side on which the larger number was presented indicated an overall numerical bisection bias toward the position of the larger number. These results replicate previous findings by Fischer (2001b) and de Hevia et al. (2006) and are compatible with those reported by Longo and Lourenco (2007). These authors observed a stronger bias in the numerical than in the non-numerical version of the line bisection task. Furthermore, the difference-based numerical bias was particularly stronger in children and elderly participants relative to young adults and middle-aged adults (see **Figures 1B, 2B**). Interestingly, elderly participants showed a strong absolute rightward bias when the larger number was presented on the right, while children showed only a relative rightward bias when the larger number was presented on the right. A tentative explanation for this finding might be that large numbers on the left are unexpected for children and thus, capture their attention there, because counting usually begins on the left with small numbers (e.g., Shaki et al., 2012). In contrast, for elderly participants all encoding is from left to right (e.g., cultural experiences such as reading and writing direction) and habitually ends on the right, focusing attention there. This is the first time that the development of the *difference-based numerical bias* was investigated in a sample with an age range from 9 to 86 years. However, we did not observe any age-related performance differences in the non-numerical version of the line bisection task, a result that is inconsistent with a general leftward bias -/rightward bias in young/old participants observed previously (Jewell and McCourt, 2000). Thus, our findings suggest that the numerical bias can be distinguished from a pure spatial bias observed in the line bisection task.

Finally and importantly, the developmental trajectory of the *difference-based numerical bias*, a SNA bound to non-directional extensions, was found to differ from that of the SNARC effect, as only the SNARC effect increased with age. These discrepant results suggest that the cognitive origins/mechanisms responsible for these two different types of SNAs, i.e., the non-directional *difference-based numerical bias* and the directional SNARC effect, seem to differ. This will be discussed in more detail in the next section.

### Association between the SNARC Effect, Bisection Bias and Age

Performance was moderately correlated in the non-numerical versions of the line bisection task. When employing flankers, however, attention orientation is additionally cued by semantic properties of these flankers, such as number magnitude (Fischer, 2001a,b). Therefore, accuracy in the line bisection task should primarily draw on the ability to select relevant spatial information in the presence of cues, which activate an association between space and number magnitude. In the presence of irrelevant and asymmetrical cues, the selection of spatial information should be more difficult and biased by cognitive properties of the cues (Fischer, 2001a; see also Fischer, 2003; Casarotti et al., 2007; Bonato et al., 2008). Therefore, participants with reduced capacity to filter out spatial information should be more sensitive to the effect of numerical cues when bisecting lines. This hypothesis was corroborated by the non-linear association between measures of the *difference-based numerical bias* and age: The *difference-based numerical bias* was stronger in children and elderly participants relative to young and middle-aged adults. This can be explained by a general weakness in inhibitory control, as a specific part of working memory (for a review see Diamond, 2013; for age related differences in inhibitory control see also Petersen et al., 2016), that is typically observed in children and elderly participants relative to young adults (Hasher and Zacks, 1988; Christ et al., 2001). As children and elderly participants should be less efficient in inhibiting the magnitude representation of numerical flankers relative to young adults, they may have showed a stronger difference-based numerical bias in the line bisection task. Working memory seems to be a more important factor for the development of the difference-based numerical bias than embodied representations of cultural experiences which result in long-term associations between number magnitude and physical space. However, executive control is involved in two-choice reaction time tasks, such as the parity judgment task, which was used in the current study to measure the SNARC effect, as well. That is, participants have to inhibit the irrelevant information, or the response activated by the irrelevant information (i.e., the spatial information associated with the target number in the case of the SNARC task). Accordingly, and irrespective of the origin of the spatial codes underlying the SNARC effect, we might expect changes in the size of the SNARC effect across the lifespan that follow the development and decline of cognitive-control abilities. This happens, for example, for the Simon effect: the size of the interference effect has been shown to decrease with age during childhood to reach adult-like levels between the sixth and tenth years of life, and then to increase again in older age (e.g., Iani et al., 2014; Kubo-Kawai and Kawai, 2010). However, in order for the interference effect to occur, the interfering information must be available. In fact, consistent evidence shows that there are SNAs in children from very early age on (e.g., de Hevia and Spelke, 2009; Girelli et al., 2009; see de Hevia et al., 2012 for a review on evidence for SNAs in toddlers). As suggested by Wood et al. (2008) there might be more than one cognitive factor which may induce age-related variability in mental associations such as the SNARC effect (i.e. practice with the association and inhibitory abilities).

Nevertheless, our account drawing on a strengthening of embodied representations reflects a more parsimonious explanation of the observed result pattern as it indicates that a single mechanism might be sufficient to account for developmental trajectories. Most importantly, however, the conclusion of two different mechanism underlying the two SNAs investigated in the current study is consistent with the fact that we did not observe a significant correlation between the difference-based numerical bias in line bisection and the SNARC effect. The latter would have been expected in case of a shared underlying mechanism incorporating influences of both embodiment and changes of working memory resources. Instead we observed linear and quadratic trends of age on the SNARC effect and the bisection bias, respectively. As such, our results indicate that the SNARC effect may more likely result from the automatic associations between number magnitude and physical space, while working memory contributes to a greater extent to the bisection bias. However, alternative explanations cannot be ruled out completely based on these data.

Taken together, the SNA-specific developmental trajectories are difficult to reconcile with the view of a single set of cognitive mechanisms underlying directional and non-directional SNAs as reflected by the SNARC effect and the numerical bisection bias – be it either embodiment or influences of working memory. However, absence of evidence does not constitute evidence of absence. Therefore, the interpretation of the present results regarding correlations between SNAs remains tentative. While the SNARC effect was associated linearly with age, the *difference-based numerical bias* presented a quadratic association with age. Regarding the SNARC effect, this indicates an increasing effect of cultural experiences (such as reading and writing direction) and thus embodied influences on long-term associations of number magnitude and physical space. In contrast, the development of the *difference-based numerical bias* in the numerical version of the line bisection task seems to be associated with changes in working memory between age groups. Even though increasingly automatic associations between number magnitude and physical space across the lifespan and age-related changes in working memory seem to be valid explanations for the differential effects of age on the SNARC effect and the bisection bias respectively, alternative interpretations cannot be ruled out completely by the present findings. Therefore, as origins of SNAs are heavily debated, alternative explanations of the present results were discussed in light of these differing accounts in an integrative approach. While the current study contributes interesting results to this ongoing discussion, future studies will be needed to pinpoint the precise cognitive mechanisms of different SNAs. On a broader level, the present results strongly suggest that associations between numbers and space, bound either to spatial directions or non-directional extensions as classified by Cipora et al. (2015), seem to have different origins.

## Author Contributions

MN, GW, and KM: analysis and interpretation of data, manuscript writing. GW: subject recruitment and data collection. LK, H-CN, and MF: Interpretation of data, manuscript writing. All authors approved the final version of manuscript.

## Conflict of Interest Statement

The authors declare that the research was conducted in the absence of any commercial or financial relationships that could be construed as a potential conflict of interest.

## Footnotes

1 Results did not change substantially when left-handed and ambidextrous persons were excluded.

2 Using the mean of all homolog items in the non-numerical version for asymmetrical and symmetrical flanker tasks as dependent variable in one univariate ANOVA and group as factor, we did not observe a significant effect either (*p* = 0.62).
